# Multispectral imaging detects gastritis consistently in mouse model and in humans

**DOI:** 10.1038/s41598-020-77145-4

**Published:** 2020-11-18

**Authors:** Thomas Bazin, Sergio Ernesto Martinez-Herrera, Aude Jobart-Malfait, Yannick Benezeth, Matthieu Boffety, Catherine Julié, Jean-François Emile, Valérie Michel, François Goudail, Eliette Touati, Franck Marzani, Dominique Lamarque

**Affiliations:** 1 Department of Gastroenterology, Université Paris Saclay/UVSQ, INSERM, Infection and Inflammation, UMR 1173, AP-HP, Hôpital Ambroise Paré, 9 avenue Charles de Gaulle, 92100 Boulogne Billancourt, France; 2 Université Paris-Saclay, Institut d’Optique Graduate School, CNRS, Laboratoire Charles Fabry, 91127 Palaiseau, France; 3 Université Paris-Saclay, UVSQ, Inserm U1173, Infection et Inflammation, Laboratory of Excellence INFLAMEX, 78180 Montigny-Le-Bretonneux, France; 4 ImViA EA7535, Université Bourgogne Franche-Comté, Dijon, France; 5 Department of Anatomical Pathology, Hôpital Ambroise Paré, AP-HP, 9 Avenue Charles de Gaulle, 92100 Boulogne-Billancourt, France; 6 Institut Pasteur, Helicobacter Pathogenesis Unit, CNRS UMR 2001, 75724 Paris cedex 15, France

**Keywords:** Medical research, Translational research, Optical spectroscopy

## Abstract

Gastritis constitutes the initial step of the gastric carcinogenesis process. Gastritis diagnosis is based on histological examination of biopsies. Non-invasive real-time methods to detect mucosal inflammation are needed. Tissue optical properties modify reemitted light, i.e. the proportion of light that is emitted by a tissue after stimulation by a light flux. Analysis of light reemitted by gastric tissue could predict the inflammatory state. The aim of our study was to investigate a potential association between reemitted light and gastric tissue inflammation. We used two models and three multispectral analysis methods available on the marketplace. We used a mouse model of *Helicobacter pylori* infection and included patients undergoing gastric endoscopy. In mice, the reemitted light was measured using a spectrometer and a multispectral camera. We also exposed patient’s gastric mucosa to specific wavelengths and analyzed reemitted light. In both mouse model and humans, modifications of reemitted light were observed around 560 nm, 600 nm and 640 nm, associated with the presence of gastritis lesions. These results pave the way for the development of improved endoscopes in order to detect real-time gastritis without the need of biopsies. This would allow a better prevention of gastric cancer alongside with cost efficient endoscopies.

## Introduction

Chronic gastritis is the consequence of *Helicobacter pylori* infection and represents the main risk factor of gastric cancer^1^. Nowadays, inflammatory and preneoplastic lesions are underdiagnosed during gastric endoscopy under white light. There are usually no macroscopically observable differences between normal mucosa and pathological tissues at the stage of inflammatory and preneoplastic lesions, using classical white-light endoscopy and even using high-definition endoscopes^2–5^. To our knowledge, there is no technology capable of exploring large areas of gastric tissue during an endoscopic examination to detect gastritis. Confocal endomicroscopy and optical coherence tomography allow real-time analysis of tissue structure on specific lesions, but do not allow scanning of large mucous surfaces at the scale of an entire digestive organ. The diagnosis of gastritis is performed from the histological analysis of biopsies, randomly collected from gastric tissue during endoscopy. However, the collection of random biopsies increases the examination time and requires extra resources^6^. Therefore, identification of gastritis by an optical device could motivate the collection of biopsy samples by the endoscopist. Conversely, the collection of biopsies could be avoided in the absence of detectable inflammation.

The chemical structure and the architecture of pathological tissues induces modifications of light absorption and reflection^7^. As a result, abnormal tissues may reflect light with a different spectrum than normal mucosa. The reflectance corresponds to the ratio between the emitted and reflected light from the tissue. Multispectral imaging is capable of acquiring images on different wavelengths of the light spectrum and measuring reflectance. It has already been used in medical applications, mainly for the identification of skin lesions^8,9^. Therefore, a minimally invasive diagnosis based on multispectral imaging could highlight histological differences in gastric tissue to facilitate the detection of patients with gastritis. This would optimize the detection of gastritis in patients, particularly those exhibiting inflammatory lesions with higher severity, leading to a better prevention of gastric cancer.

Using the mouse model of *H. pylori* infection to generate inflammatory gastric lesions and, in parallel, endoscopy examination in patients, the aim of our study was to determine the variation in reflectance of gastric tissue and its relationships with inflammatory lesions at different stages of gastritis.

## Methods

### Mouse model

#### Bacterial strains and growth conditions

The *H. pylori* strain SS1, able to colonize the mouse gastric mucosa for long periods^10^, was used in this study. Bacteria were grown on blood agar base 2 (Oxoid Lyon, France) plates supplemented with 10% defibrinated horse blood (bioMérieux, Marcy L’Etoile, France) and an antibiotic-antifungal mixture. The plates were incubated at 37 °C for 24–48 h under microaerobic conditions (7% O_2_, 10% CO_2_; Anoxomat system).

#### Mouse infection

Mouse experiments were carried out in strict accordance with European recommendations. The protocol has been approved by the Committee of Central Animal Facility Board, the Ethic committee on animal experimentation of the Institut Pasteur (Ref 2013-0051) and the French Ministry of Higher Education and Research (Ref 00317.02).

Five-weeks old NMRI female mice (Charles River Laboratories; France) were housed in polycarbonate cages and acclimatized for 1 week before starting the experiments. In total 24 mice were included in the present study: 12 *H. pylori* SS1-infected mice, which were orogastrically inoculated with 150 µl of a suspension of bacteria (10^8^ colony forming unit (CFU)/ml) and 12 non-infected mice which received 150 µl of peptone broth. After 1, 3, 7 and 12 months, 3 infected and 3 non-infected mice were sacrificed, their stomach isolated and fragments containing antrum and fundus parts were used for histology and reflectance spectra analysis of the gastric inflammatory lesions. In addition, *H. pylori* gastric colonization was quantified as previously described^11^.

#### Histological analysis

Stomach samples from non-infected and infected mice were fixed in RCL2 (Alphelys, France) and embedded in low-melting-point paraffin wax (Poly Ethylene Glycol Distearate; Sigma, USA). Four µm-thick sections were stained by hematoxylin and eosin treatment (H&E) and examined blindly for neutrophil infiltration and mononuclear cell infiltration, which were semi-quantitatively evaluated as previously described^12,13^, based on a scoring system with four severity grades (0 = none, 1: mild, 2: moderate, 3: severe) according to the Updated Sydney System^14^.

#### Tissue reflectance

The reflectance of the mucosa was measured using two different methods: (a) a spectrometer Konica Minolta CM-2600d, which has an integrated source of light and retrieves the reflectance of the measured surface (6 mm diameter disk) from 400 to 740 nm, at 10 nm increments (Fig. 3A); (b) a multispectral camera Flux Data FD1665, retrieving 7 monoband images from 480 to 810 nm (width from 30 to 100 nm), from which 6 are in the visible spectrum and 1 in the near infrared. This camera has 3 independent sensors for the acquisition. The acquisition surface was also 6 mm disks. Acquisitions were performed using a dedicated software from the University of Bourgogne. The light source was a Xenon lamp. In order to reduce the specularity, an angle of 45° was configured between the light source and the multispectral camera. In addition, two linear polarizers were included, one for the light source and the other for the multispectral camera configured in cross polarization. A white calibration was performed before each set of acquisition.

### Human

#### Subjects

We included patients referred to the endoscopic unit of Ambroise Paré hospital, Boulogne-Billancourt, France. Patients were eligible if they had been scheduled for gastroendoscopy under general anesthesia and had given their written informed consent, as approved by the Comité de Protection des Personnes Sud-Est III ethics committee on June 2019 (registration number: 19.06.21.76520). The study was conducted according to the World Medical Association Declaration of Helsinki. Patients with chronic gastritis (n = 8) were enrolled from the cohort GASTRIMED (ClinicalTrials.gov identifier: NCT02325323) funded by the French Gastroenterology Society (SNFGE), including adults which had a gastroscopy in the last 6 months with antral and gastric body biopsies. The aim of this cohort is to study the association between changes in the gastric mucosa in chronic gastritis and the onset of cancer.

#### Procedure

##### Multispectral acquisition

During gastroendoscopic examination, the endoscope was focused on the antrum. Then we used the multispectral acquisition system as described in^15^. A xenon lamp was used as the light source, since it provides constant stable illumination, which can be easily filtered in order to provide illumination at different wavelengths. In order to acquire multispectral images, the light was filtered by a rotating filter wheel. Thanks to the sequential rotation of filters, the tissue was illuminated with a specific wavelength for a short period of time. The filter wheel contained 5 filters centered at 440 nm, 520 nm, 560 nm, 600 nm and 640 nm, with a full width at half maximum of 80 nm. The acquisition time for one full rotation of the filter wheel was slightly more than one second. At the same time, images were collected by the native camera from the gastroendoscope (Olympus Exera II) with the magnification option disabled.

This device allowed us to capture a multispectral image from the antrum after being illuminated sequentially with 5 different wavelengths. The time allocated for the collection of multispectral images was 30 s, which was enough to perform multiple acquisitions.

##### Biopsy collection

After multispectral acquisition we switched to the normal white light examination mode in order to perform systematic biopsy collection from the antrum and the corpus, according to standard protocol. These biopsies were placed in 10% formalin and embedded in paraffin. Transversal gastric tissue section samples were stained by H&E for routine histological analysis. In addition, polymerase chain reaction (PCR) and immunochemistry using PFLEX Polyclonal Rabbit antibody (Dako) were performed to detect *H. pylori* infection. Biopsies were scored for the severity of inflammatory lesions according to the Updated Sydney System^14^. The histopathological diagnosis was performed by a senior pathologist (CJ).

##### Image and spectral treatment

Each gastroendoscopic video, acquired during endoscopy procedure, was divided in sequences of multispectral images. Each multispectral image contained 5 images (640 × 480 pixels, 8 bits), one for each wavelength used for illumination.

The gastric wall could perform some movements during the acquisition. In order to correct these micromovements of the tissue, we used multispectral images where the images at each wavelength were sharp and showed low movements between them. Next, the registered multispectral images were thresholded in order to remove specular areas. In addition, the user manually selected areas of tissue from the image which were correctly exposed in order to remove artifacts such as bubbles of gastric juice, borders of folds of gastric tissue and the pylorus sphincter. Then, spectra were standardized by mathematical normalization to avoid the influence of the distance and angle between the tissue and the gastroendoscope.

### Statistical analysis

The spectra from the multispectral images were randomly sampled from the observed areas of gastric tissue. The sample consists of 20 areas of the image of size 5 × 5 pixels from each multispectral image, which were averaged in order to retrieve a representative spectrum for each multispectral image. This procedure was repeated 10 times for each multispectral image.

After separation into two clinical groups (normal *vs* gastritis) based on histology analysis, we computed a reference spectrum for the normalized data of the control group, as the median response at each captured wavelength. Then, each patient from the gastritis group was compared to the control group in order to measure the percentage of variation.

In order to compare the differences between the spectra from the two groups, Wilcoxon-Mann–Whitney test was performed with Bonferroni correction (posthoc) on the normalized spectra; p values < 0.05 were considered statistically significant.

Raw data are available on request.

### Ethical approval

This study was approved by the Comité de Protection des Personnes Sud-Est III ethics committee in June 2019 (registration number: 19.06.21.76520). A written informed consent was obtained from all the patients involved in the study.

## Results

### Mouse

#### Histological analysis

**Figure 1:**
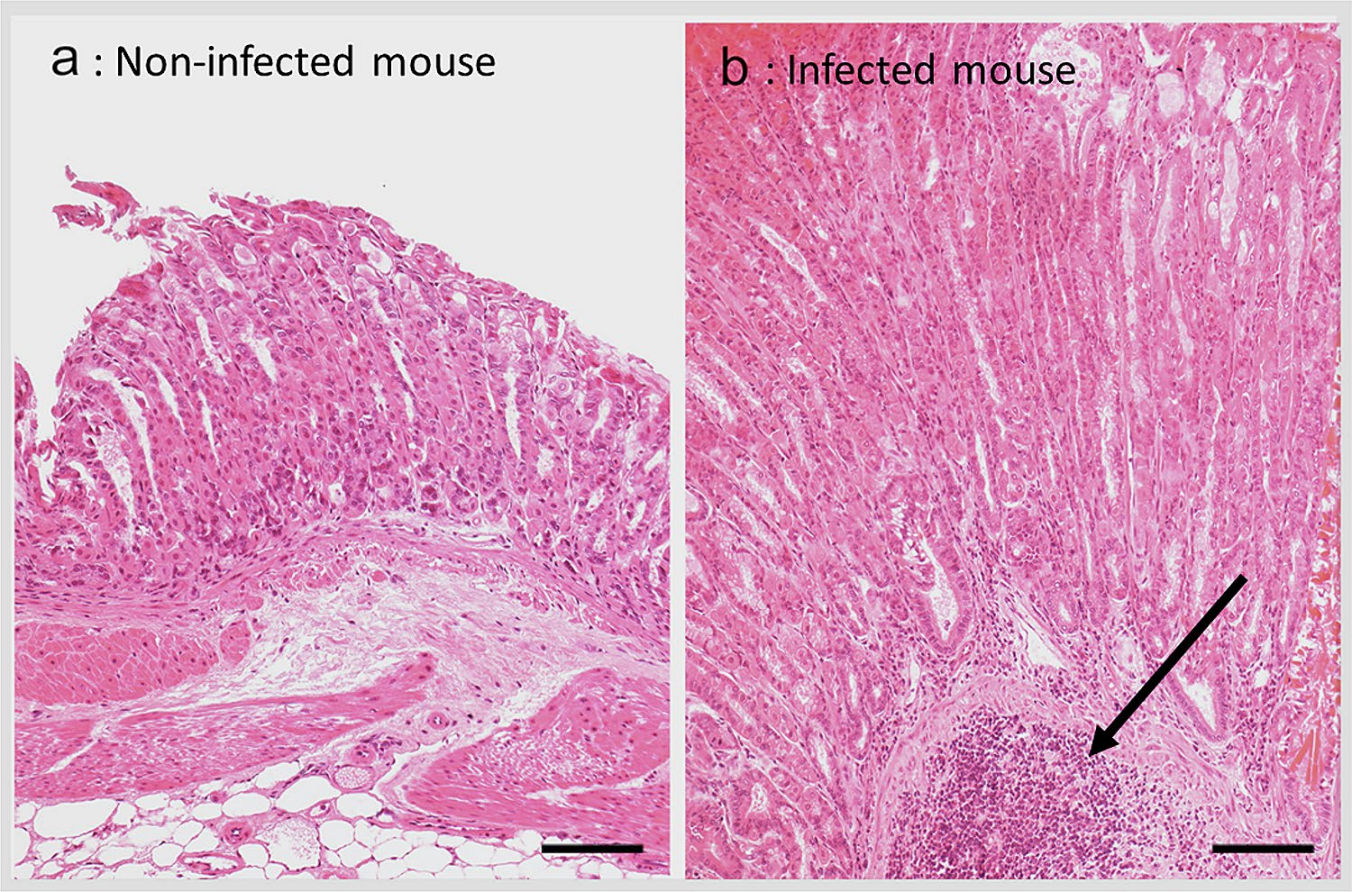
Gastric histology at 12 months in non-infected control (n = 12, **a**) and *H. pylori* infected mice (n = 12, **b**). Mononuclear cell infiltration and aggregates (arrow) are easily visible in the infected mucosa (scale bar 100 µm) (Photo L. Fiette, Institut Pasteur).

**Figure 2:**
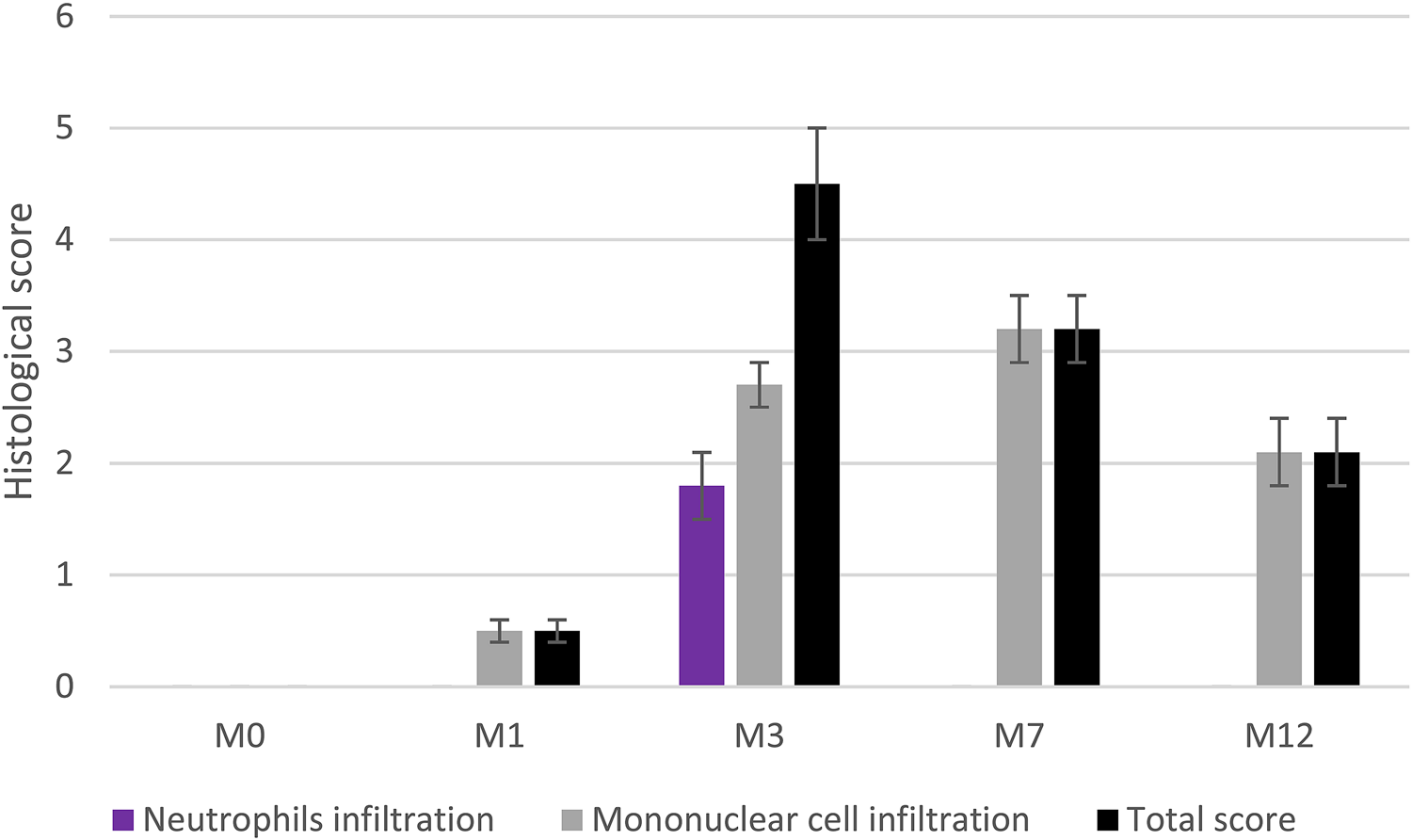
Evolution of mouse gastric mucosa inflammation after *H. pylori* infection. Values are means of histological score ± SD.

The gastric mucosa of all infected mice was efficiently colonized by the *H. pylori* strain SS1 at each time-point. Mean of CFU per gram of gastric tissue ± SD was 2 ± 3, 0.8 ± 1.5, 0.2 ± 0.5, 0.2 ± 20 at month (M)1, M3, M7 and M12, respectively. No *H. pylori* colonization was observed in the control groups. Histological modifications were similar to those observed in previously published work^16^. After 1 month of infection (M1), gastric histological analysis showed low mononuclear cell infiltration without any polynuclear cell infiltrate [total score 0.5 ± 0.1 (mean ± SD)]. At M3 a dense polynuclear infiltrate was observed, corresponding to a mild inflammation (total score 4.5 ± 0.5). At M7 and M12 the infiltrate was only mononuclear, corresponding to a moderate (total score 3.2 ± 0.3) and mild (total score 2.1 ± 0.3) chronic inflammation at M7 and M12, respectively (Figs. 1b, 2). No histological inflammation was found in the control groups (Fig. 1a). Histological images at M12 can be found online as Supplementary Figures S1–S6 for infected mice at M12, and Supplementary Figure S7 for non-infected mice at M12.

#### Spectrometer

**Figure 3:**
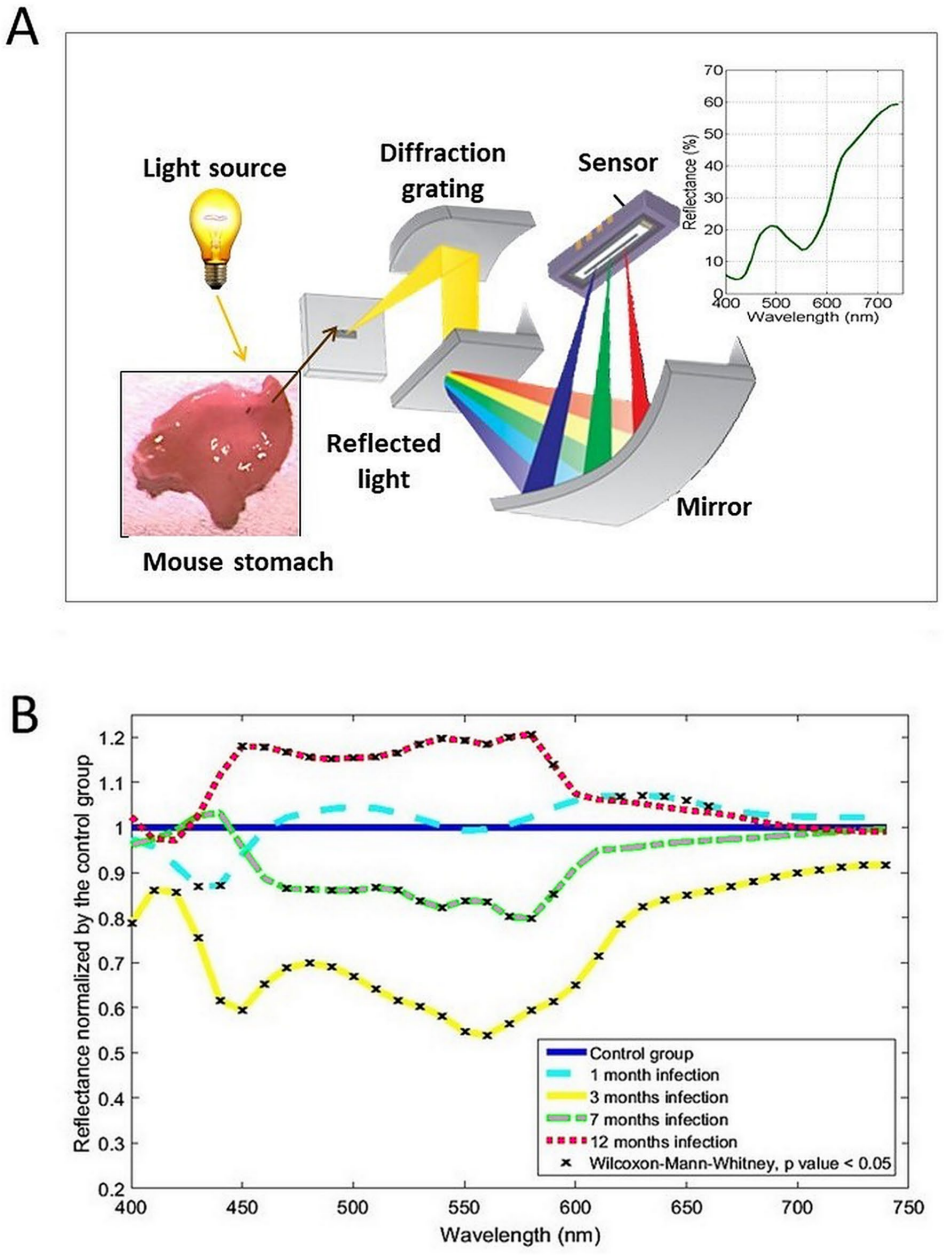
(**A**) Spectrometer setup and principle. The light reflected from the tissue is decomposed and measured all along the visual light spectrum. (**B**) Reflectance variations, normalized by the control group, measured using the spectrometer, at each time point (10 nm increment).

The acquisition spectra of the infected stomachs at each time-points, normalized to the corresponding control stomachs are presented in Fig. 3B. Of note, reflectance variations were not significant in controls. We identified 3 bands of wavelengths which were significantly modified between the two groups at more than one time-point. These bands correspond to 430–450 nm, 470–590 nm and 620–660 nm. In the infected groups, the reflectance band between 430 and 450 nm was significantly reduced at M1 and M3. The reflectance band between 470 and 590 nm was significantly reduced at M3 and M7 and increased at M12. In addition, the reflectance band between 620 and 660 nm was significantly increased at M1 and reduced at M3. Of note, spectrum intensity was significantly reduced at all wavelengths at M3.

#### Multispectral camera

**Figure 4:**
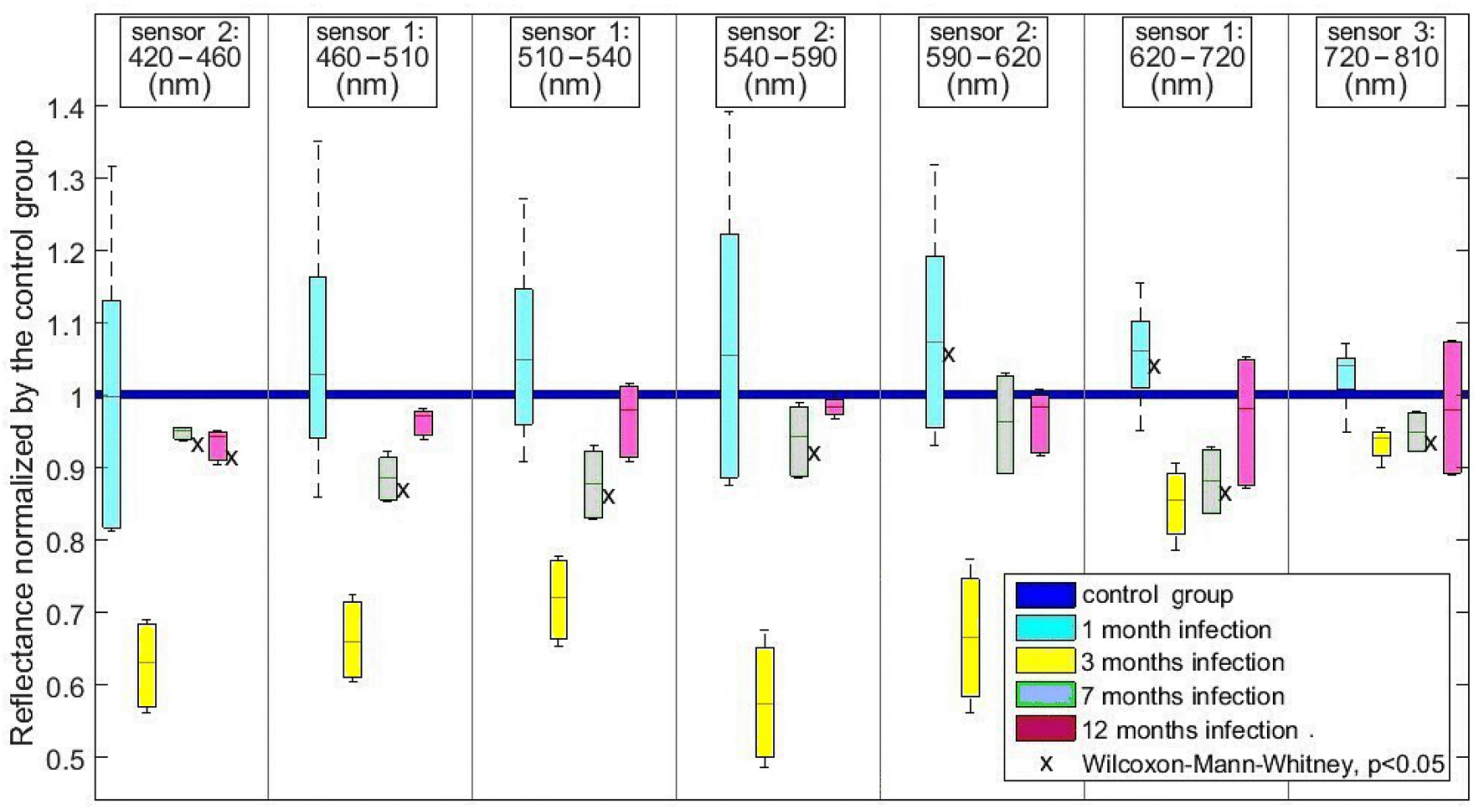
Reflectance variations obtained using the multispectral camera and normalized on the non-infected control group, for each monoband image and at each time-point of *H. pylori* infection.

Results are presented in Fig. 4. Again, reflectance variations were not significant in controls. The reflectance band between 420 and 460 nm was reduced at M7 and M12. The reflectance band between 510 and 540 nm was reduced at M7. The reflectance band between 540 and 590 nm was reduced at M7. The reflectance band between 590 and 620 nm was increased at M1. The reflectance band between 620 and 720 nm was increased at M1 and reduced at M7. The reflectance band between 720 and 810 nm was reduced at M7.

Whatever the reflectance acquisition method used, no correlation was found between the level of *H. pylori* gastric colonization, the score grading of the inflammatory lesions and the intensity of the spectra observed.

### Human

#### Clinical and pathological characteristics of patients

The present study was performed over a period of one year between July 2019 and February 2020. A written informed consent was obtained from all the patients involved in the study. We collected a total of 62 gastroendoscopic videos from 62 patients included in GASTRIMED cohort. We only kept 25 videos which did not show any evidence of visible lesions under white light, in order to avoid re-emitted light modification due to macroscopic abnormalities such as gastric atrophy or ulcers. The patients were grouped according to one of the two clinical conditions based on histological results: control group or gastritis. The control group presented only rare mononuclear cells in the mucosa (Sydney score = 0) and no *H. pylori* infection. On the other hand, the pathological group were positive for *H. pylori* infection, as assessed by immunohistochemistry or by specific PCR, and inflammatory infiltrate including mononuclear and polynuclear cells [Sydney score = 1.1 ± 1.0 (mean ± SD)]. In Table 1 are presented the clinical features of the two groups. There were no significant differences in gender or age between the groups.

**Table 1:** Patients characteristics.

	N	Age (±SEM)	Gender ratio (H:F)
Gastritis group	8	54.9 (± 5.8)	3:1
Control group	17	59.6 (± 2.6)	0.7:1

#### Multispectral analysis

The mean number of images studied was 4.6 (± 1.1) in gastritis group and 5.3 (± 0.9) in control group (mean ± SEM, NS). The mean pixel number analyzed was 23,125 (± 5664) in the gastritis group and 26,470 (± 4613) in the control group (mean ± SEM, NS) (see Method section, Image and spectral treatment sub-section for the description of image pre-processing).

**Figure 5:**
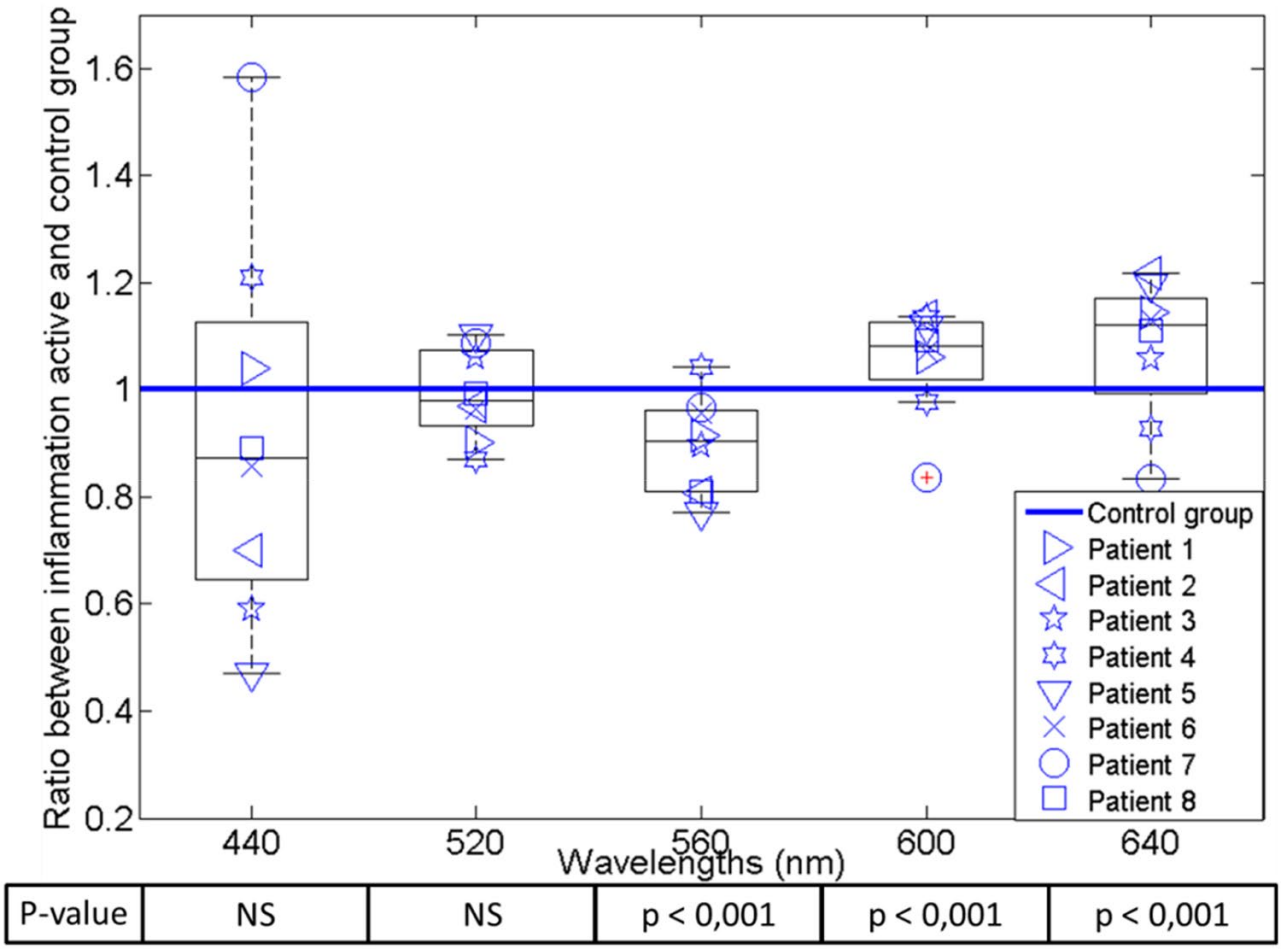
Ratio of variation in the spectrum from patients with active inflammation compared with the reference spectrum from the control group. P values were obtained using Wilcoxon–Mann–Whitney test.

The spectra analysis in Fig. 5 showed that the intensity of wavelengths at 560 nm, 600 nm and 640 nm was statistically different for active gastritis patients, compared to the normal group.

The wavelength of 560 nm showed a significant decrement of the reflected light with respect to the reference of the control group. On the other hand, we observed a significant increment of the reflected light at the wavelengths of 600 nm and 640 nm in the gastritis group.

No correlation was found between reflectance variation and Sydney scores.

**Figure 6:**
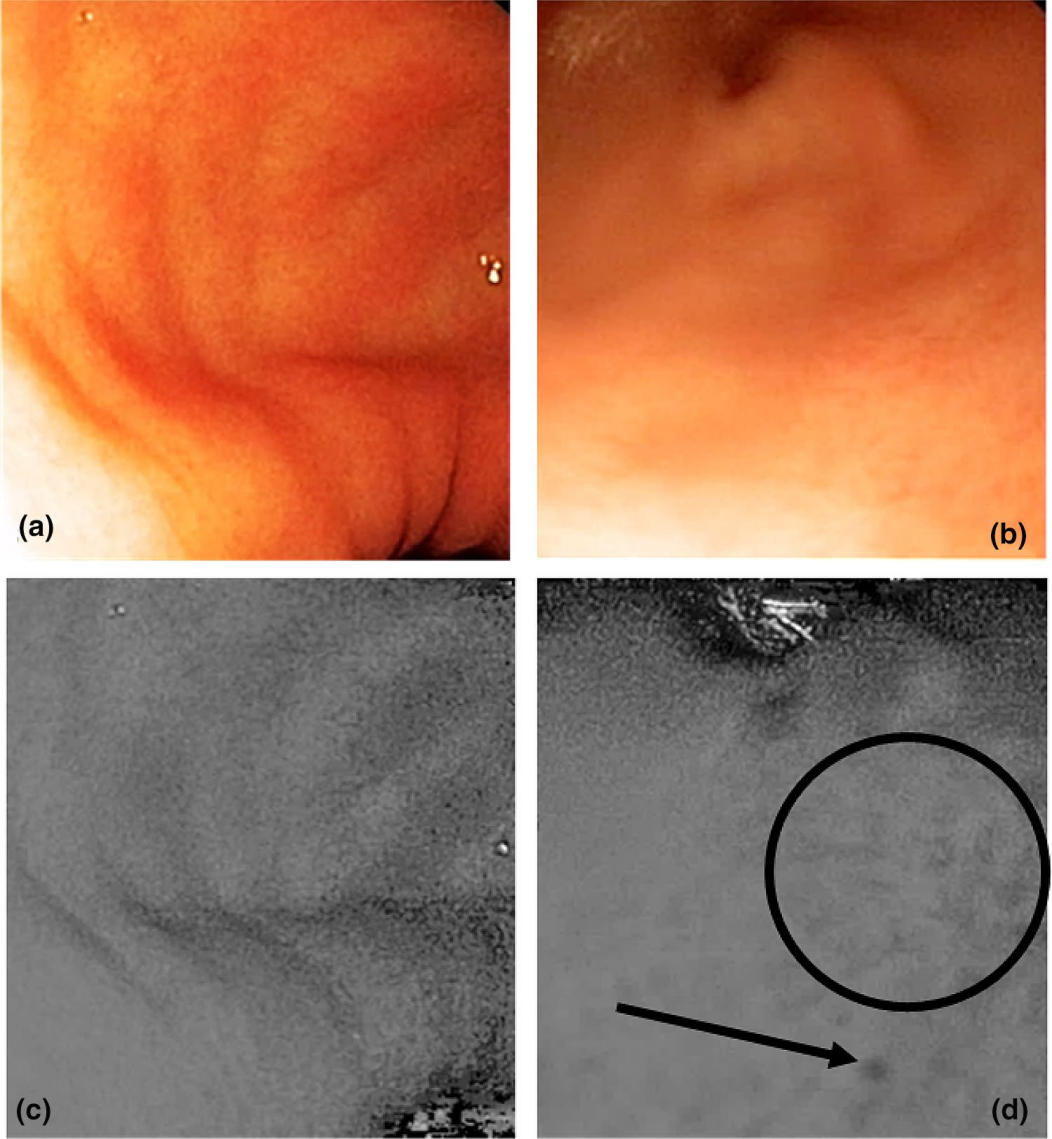
Images under white light from the control group (**a**) and gastritis patients (**b**). In the band at 560 nm, we observed that the tissue from the control group (**c**) was smooth, compared to the gastritis tissue (**d**) which presented black spots (arrow) and a non-uniform surface. The band at 560 nm highlighted areas with a wavy appearance (black circle) in the patients with inflammation (**d**).

The multispectral images were observed by senior endoscopists (DL and TB). As it was stated in the protocol, we were not able to observe differences between images under white light from control or gastritis patients, as illustrated in Fig. 6a,b. Consequently, the differences observed were only due to illumination with specific wavelengths. The band at 560 nm enhanced the contrast and highlighted areas with a wavy appearance in patients with active inflammation compared to controls as shown in Fig. 6c,d. This showed evidence of texture modification which was not visible under white light. In contrast, the images from subjects in the control group showed a smooth uniform mucosal surface.

## Discussion

To our knowledge, the present study is the first that analyzes in parallel the reflected light properties of gastric tissue inflammation related to *H. pylori* infection in the mouse model, and multispectral in vivo images of the stomach in humans.

In mouse, the variations in reflectance differ according to the timepoints: the values ​​generally tend to increase at M1 (and at M12 for the spectrometer), and to decrease at other timepoints. We do not have data to precisely explain these variations, but the differences in histological sub-scores could be an explanation. For example, neutrophil infiltration could explain the increase in reflectance at M3. Similarly, at 12 months, the large aggregates of inflammatory cells observed could explain the change in reflectance.

Our data from the spectrometer and multispectral camera show statistically significant changes in reflectance at different stages of the inflammatory lesions induced by *H. pylori* in the gastric mucosa of mice infected from 1 to 12 months*.* Importantly, the sensitivity of reflectance analysis is such that it can measure reflectance variations even only 1 month after *H. pylori* infection, where no inflammatory lesions are identified by histological analysis (Sydney score less than one). Tissue inflammation is not limited to cellular infiltrate. Other phenomena such as hemoglobin saturation, water concentration levels and cytoskeleton rearrangements are involved in inflammatory process and can play a role in reflectance modifications^17^.

In mice, whatever the method of light analysis used, we observed that the reflectance between 620 and 660 nm was statistically different after 1 and 3 months of infection. The changes at these wavelengths could be explained by differences in blood oxygenation, as oxyhemoglobin reflectance is higher between 600 and 700 nm in comparison with deoxy-hemoglobin^18^. At 12 months, the reflectance between 450 and 590 nm was significantly different between the infected and non-infected groups; these modifications could be related to size changes of the scatters and again the oxygen saturation of the tissue^19^.

The resulting tendency between the two methods in mice is identical (the variations go in the same direction), except at M12, where the spectrometer values are globally higher than in the control group, and the multispectral camera values are globally lower. This could be related to the uneven spatial distribution of lymphocyte aggregates which is only observable at M12. As reflectance measurements are based on 6 mm disks, they could simply miss lymphocytes aggregates.

Spectrometer is the gold standard of light intensity measurement, with small increments and high sensitivity. The spectral resolution of the multispectral camera is significantly lower than that of the spectrometer. But camera spatial resolution is higher, and its wide range of acquisition allows the analysis of near infrared wavebands, unlike the spectrometer we used. Aiming to extrapolate our results to endoscopic methods for surveillance of large surfaces of tissues, results from the multispectral camera are of peculiar interest, especially since they are coherent with those of the spectrometer. Of note, multispectral camera results in mice did not show any statistical significance at M3 despite high differences, probably due to high variability.

In humans, changes in light based on tissue properties have been shown to be useful for diagnosis in human health^7,20^. New technology, such as narrow band imaging (NBI, Olympus), takes advantage of the tissue response at different wavelengths. It provides qualitative images that show clues of the sub-epithelial capillary network, by measuring the peaks of absorption of hemoglobin. However, the inflammatory cell infiltration cannot be detected as there is no significant change in the endoscopic appearance during the gastritis condition. In contrast with these systems, the optical device that we used during patients endoscopies analyzed more wavelengths and could be used for diagnosis by identifying inflammation-related quantitative changes in the tissue.

In humans, we observed that reflectance wavelengths at 560 nm, 600 nm and 640 nm are different in gastritis patients. Areas with a wavy appearance were highlighted at 560 nm; this wavelength is related to the absorption peak of hemoglobin and correlated to micro vessels density^21,22^. The reflectance changes observed at 600 nm and 640 nm could be linked with variation of cytoskeleton components, as collagen networks involved in tissue repair may also change the reflectance above 600 nm^23–26^. It should be noted that we observed a similar decrease of reflectance at 560 nm between infected mice at M7 and patients with gastritis. This observation suggests that similar events occurring both in mice and humans could account for the observed spectra. However, the identification of the physiological and biochemical origin of the mechanisms which, during inflammation, are responsible for the variation in reflectance need to be further investigated.

Interestingly, our method does not require any external marker, unlike other techniques using a marker that is injected or applied locally^27^. The safety and accessibility of this method are therefore guaranteed.

In this exploratory study, using multispectral imaging, we have robustly shown in the mouse model and in humans that gastritis mucosa exhibits modifications of reemitted light around 560 nm, 600 nm and 640 nm. These promising results pave the way for the development of improved endoscopes with tailored virtual chromoendoscopic properties to detect gastritis. These three wavelengths could thus be used as biomarkers of gastritis and allow real-time optical biopsy obtention, leading to a better prevention of gastric cancer with cost efficient endoscopies.

## Supplementary information

Supplementary Information 1.
