# Author Correction: Multispectral imaging detects gastritis consistently in mouse model and in humans

**DOI:** 10.1038/s41598-021-94899-7

**Published:** 2021-08-17

**Authors:** Thomas Bazin, Sergio Ernesto Martinez‑Herrera, Aude Jobart‑Malfait, Yannick Benezeth, Matthieu Boffety, Catherine Julié, Jean‑François Emile, Valérie Michel, François Goudail, Eliette Touati, Franck Marzani, Dominique Lamarque

**Affiliations:** 1 Department of Gastroenterology, Université Paris Saclay/UVSQ, INSERM, Infection and Inflammation, UMR 1173, AP-HP, Hôpital Ambroise Paré, 9 avenue Charles de Gaulle, 92100 Boulogne Billancourt, France; 2 Université Paris-Saclay, Institut d’Optique Graduate School, CNRS, Laboratoire Charles Fabry, 91127 Palaiseau, France; 3 Université Paris-Saclay, UVSQ, Inserm U1173, Infection et Inflammation, Laboratory of Excellence INFLAMEX, 78180 Montigny‑Le‑Bretonneux, France; 4 ImViA EA7535, Université Bourgogne Franche-Comté, Dijon, France; 5 Department of Anatomical Pathology, Hôpital Ambroise Paré, AP-HP, 9 Avenue Charles de Gaulle, 92100 Boulogne‑Billancourt, France; 6 Institut Pasteur, Helicobacter Pathogenesis Unit, CNRS UMR 2001, 75724 Paris cedex 15, France

Correction to: *Scientific Reports* https://doi.org/10.1038/s41598-020-77145-4, published online 18 November 2020

The Supplementary Information 1 file published with this Article contained an error in the captions of Figures S1 to S6, where “non-infected mice” now reads “infected mice”.

The original Supplementary Information 1 file is provided below.

This error has now been corrected in the Supplementary Information 1 file that accompanies the original Article.

## Supplementary Information

Supplementary Information 1.

