# Supplementary material for: Multispectral imaging detects gastritis consistently in mouse model and in humans
Source: Sci Rep. 2020 Nov 18;10:20047. doi: 10.1038/s41598-020-77145-4 (PMC7674504; doi:10.1038/s41598-020-77145-4)
Supplement: Supplementary file 1 — Supplementary Information 1. [file 41598_2020_77145_MOESM1_ESM.docx]

Multispectral imaging detects gastritis consistently in mouse model and in humans

Thomas Bazin^1)*^, Sergio Ernesto Martinez-Herrera^2,3)^, Aude Jobart-Malfait^3)^, Yannick Benezeth^4)^, Matthieu Boffety^2)^, Catherine Julié^5)^, Jean-François Emile^5)^, Valérie Michel^6)^, François Goudail^2)^, Eliette Touati^6)^, Franck Marzani^4)^, Dominique Lamarque^1)^

1) Université Paris Saclay/UVSQ, INSERM, Infection and Inflammation, UMR 1173, AP-HP, Hôpital Ambroise Paré, Department of Gastroenterology, F92100, Boulogne-Billancourt, France

2) Université Paris-Saclay, Institut d'Optique Graduate School, CNRS, Laboratoire Charles Fabry, 91127, Palaiseau, France

3) Université Paris-Saclay, UVSQ, Inserm U1173, Infection et inflammation, Laboratory of Excellence INFLAMEX, 78180, Montigny-Le-Bretonneux, France

4) ImViA EA7535, Univ. Bourgogne Franche-Comté, Dijon, France

5) Hôpital Ambroise Paré, AP-HP, 9 Avenue Charles de Gaulle, Department of Anatomical Pathology, F92100, Boulogne-Billancourt, France

6) Institut Pasteur, Helicobacter Pathogenesis Unit, CNRS UMR 2001, F75724, Paris cedex 15, France

# Supplementary Figures


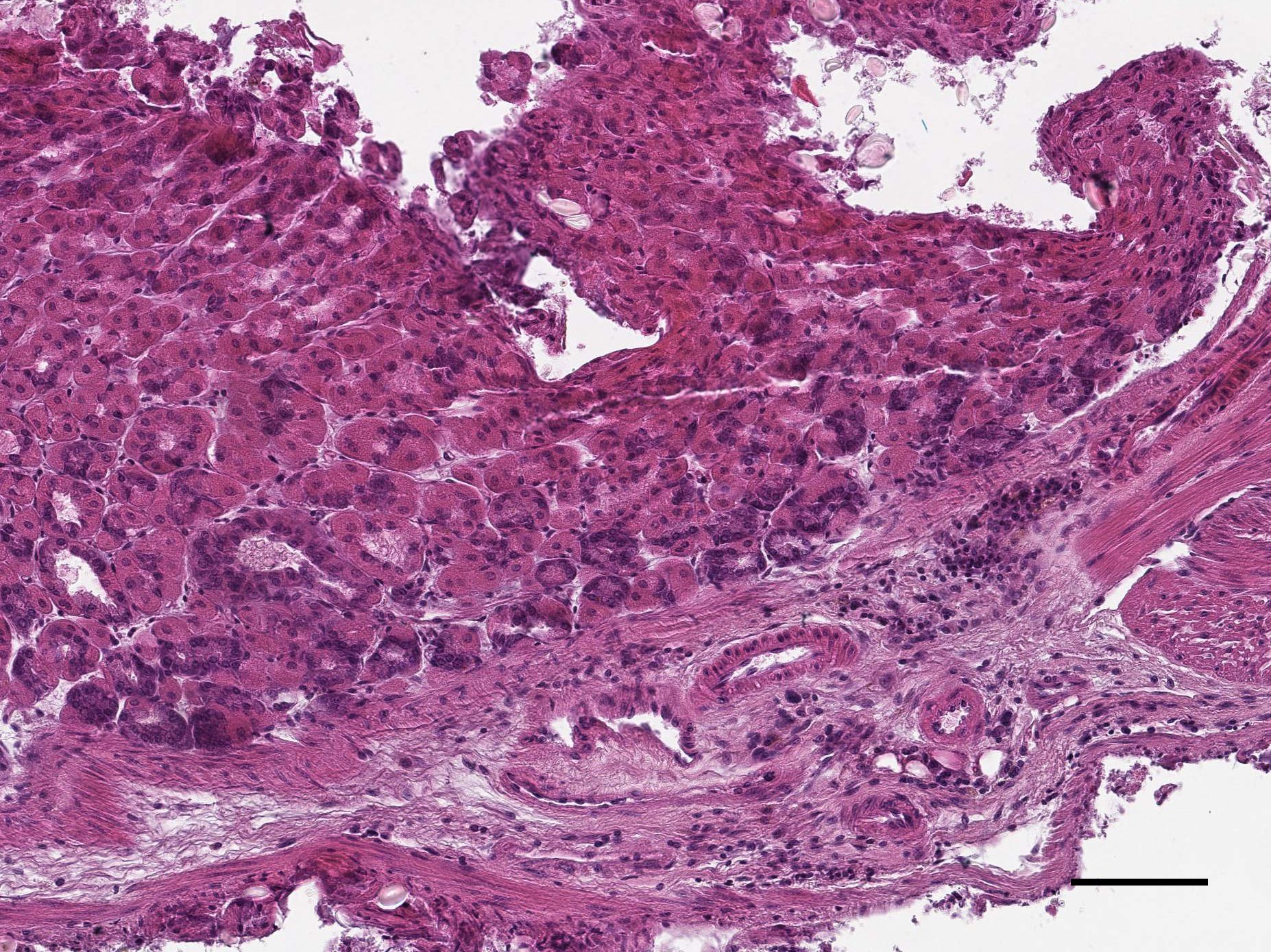
 Figure S1: infected mice at M12 (scale bar: 100µm)
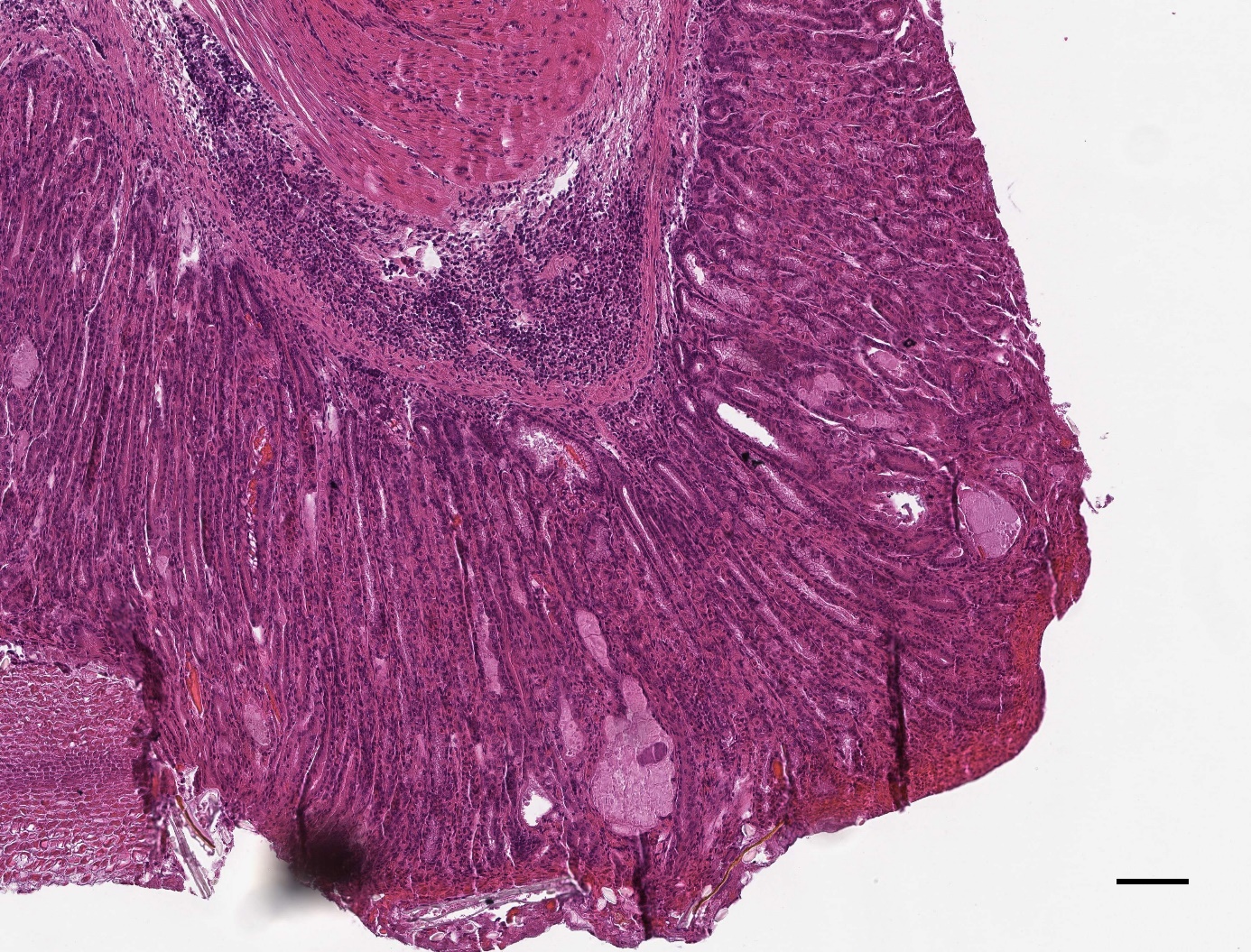
 Figure S2: infected mice at M12 (scale bar: 100µm)
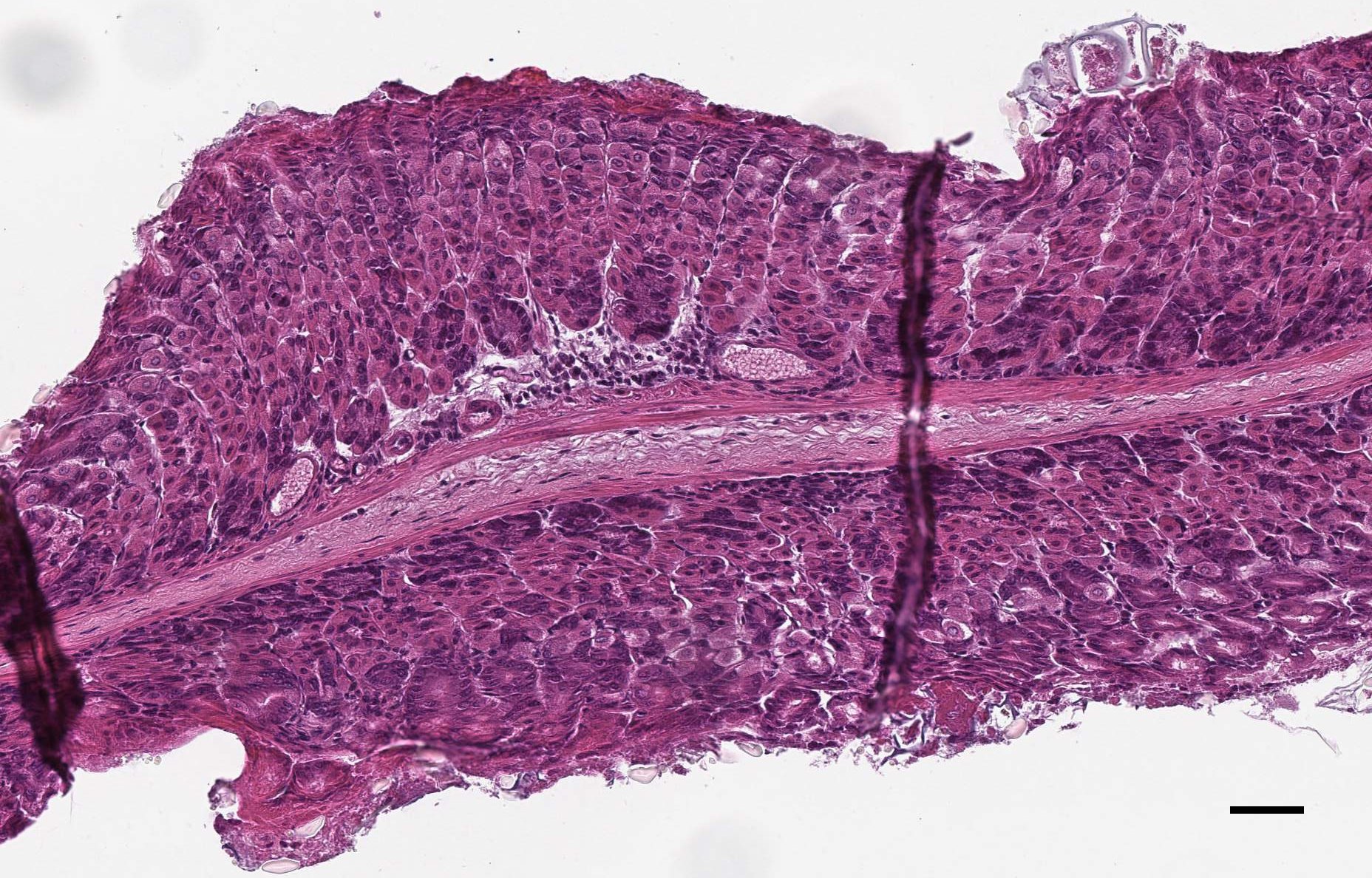
 Figure S3: infected mice at M12 (scale bar: 100µm)
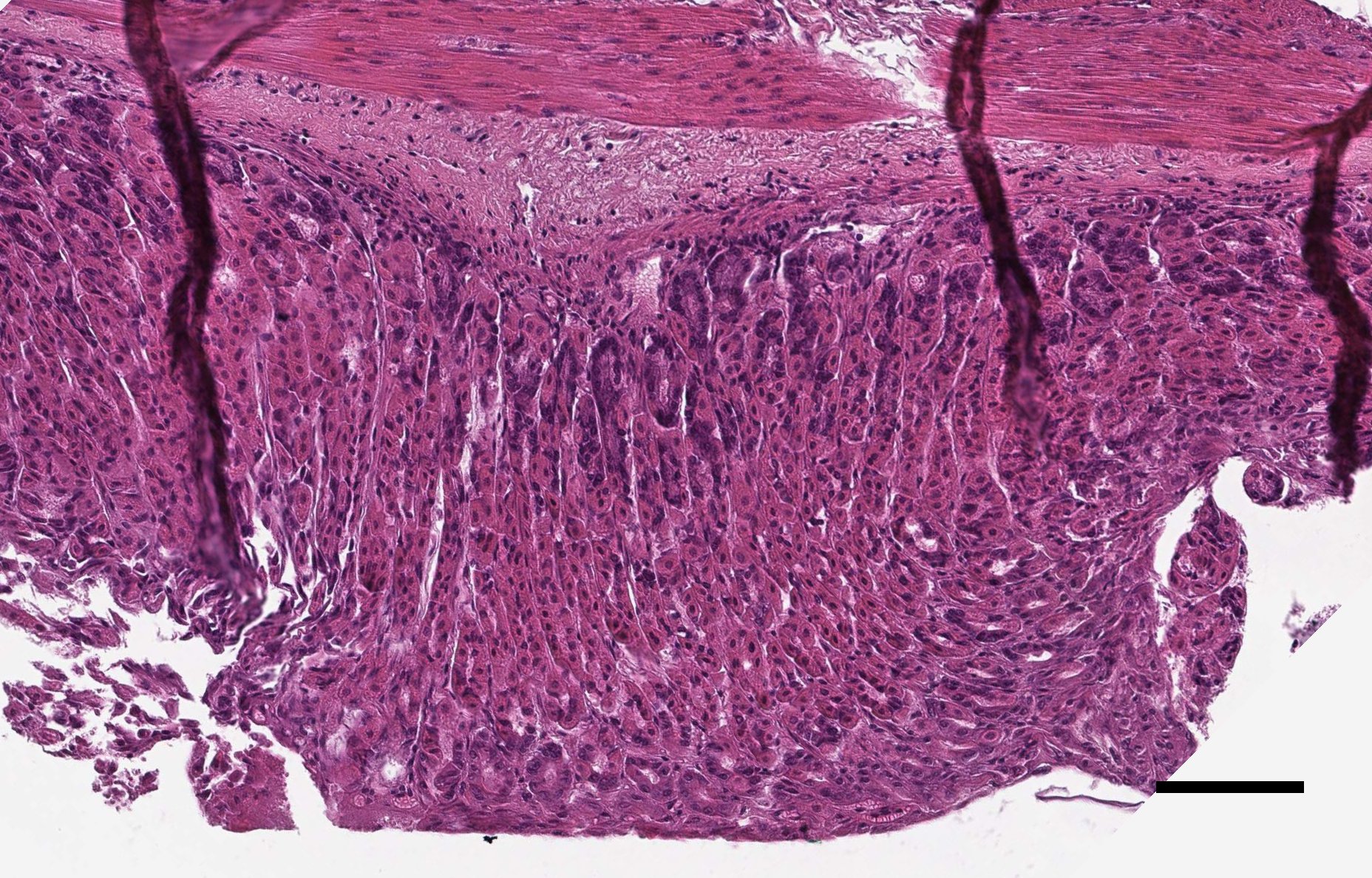
 Figure S4: infected mice at M12 (scale bar: 100µm)
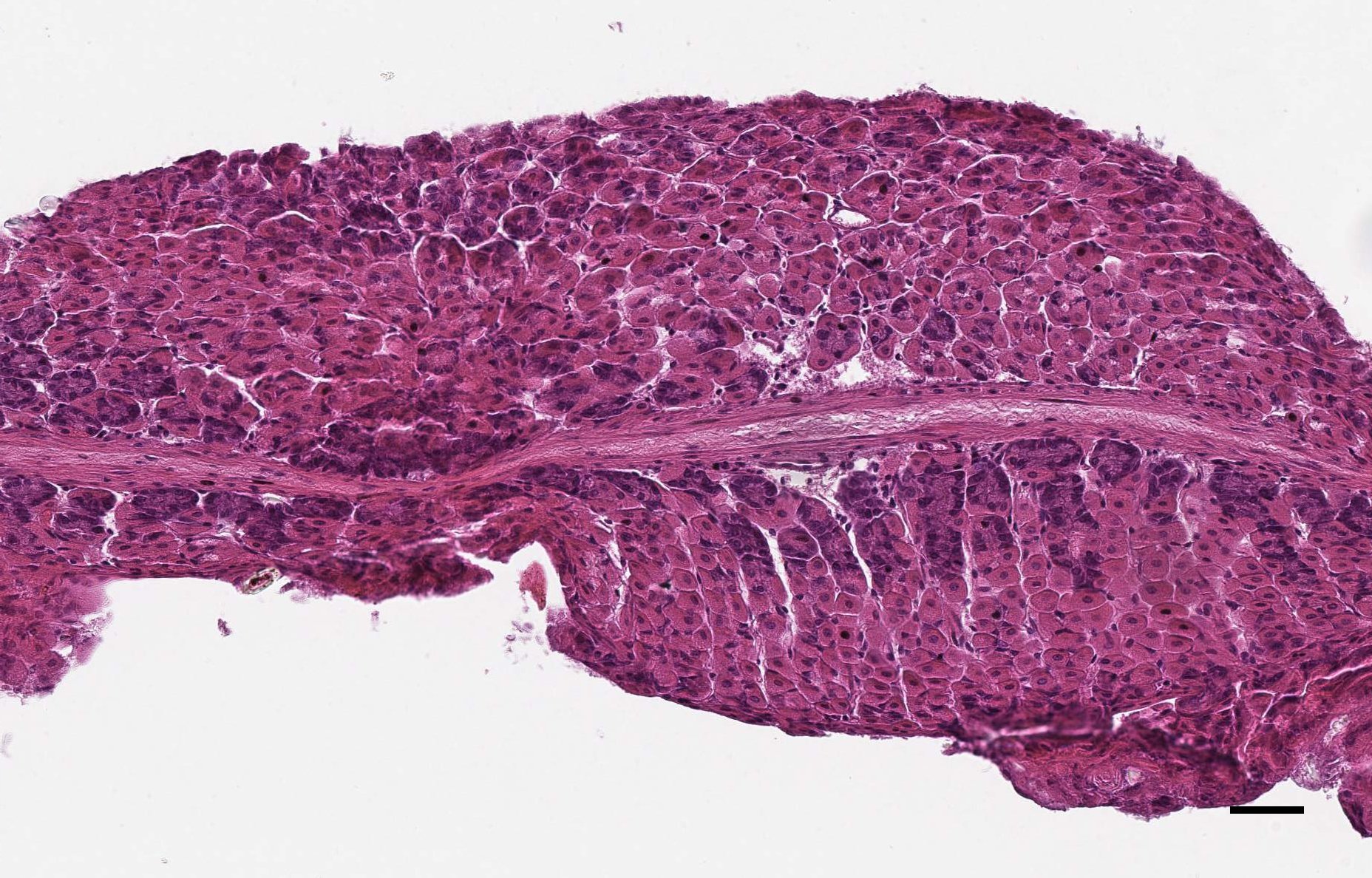
 Figure S5: infected mice at M12 (scale bar: 100µm)
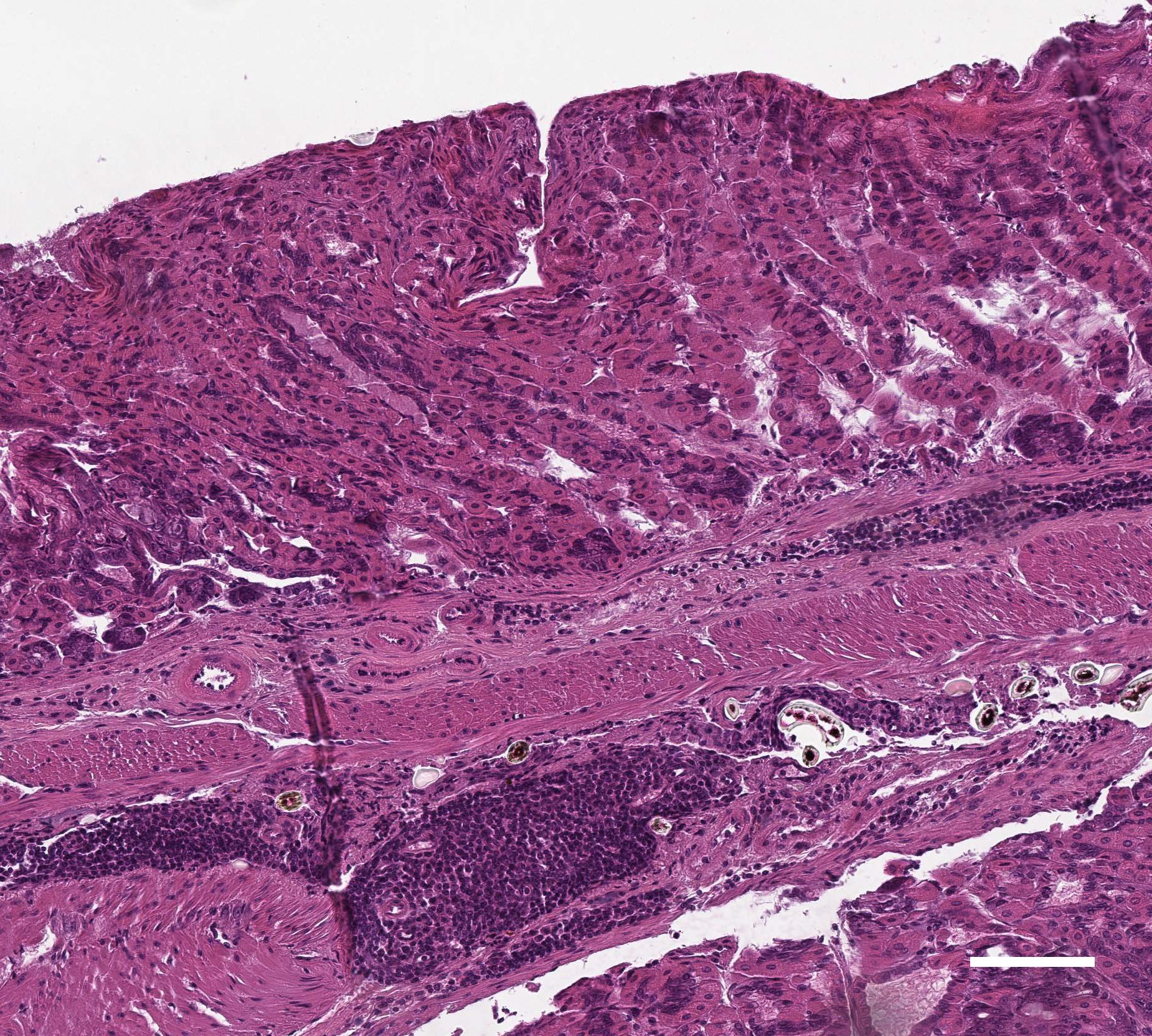
 Figure S6: infected mice at M12 (scale bar: 100µm)
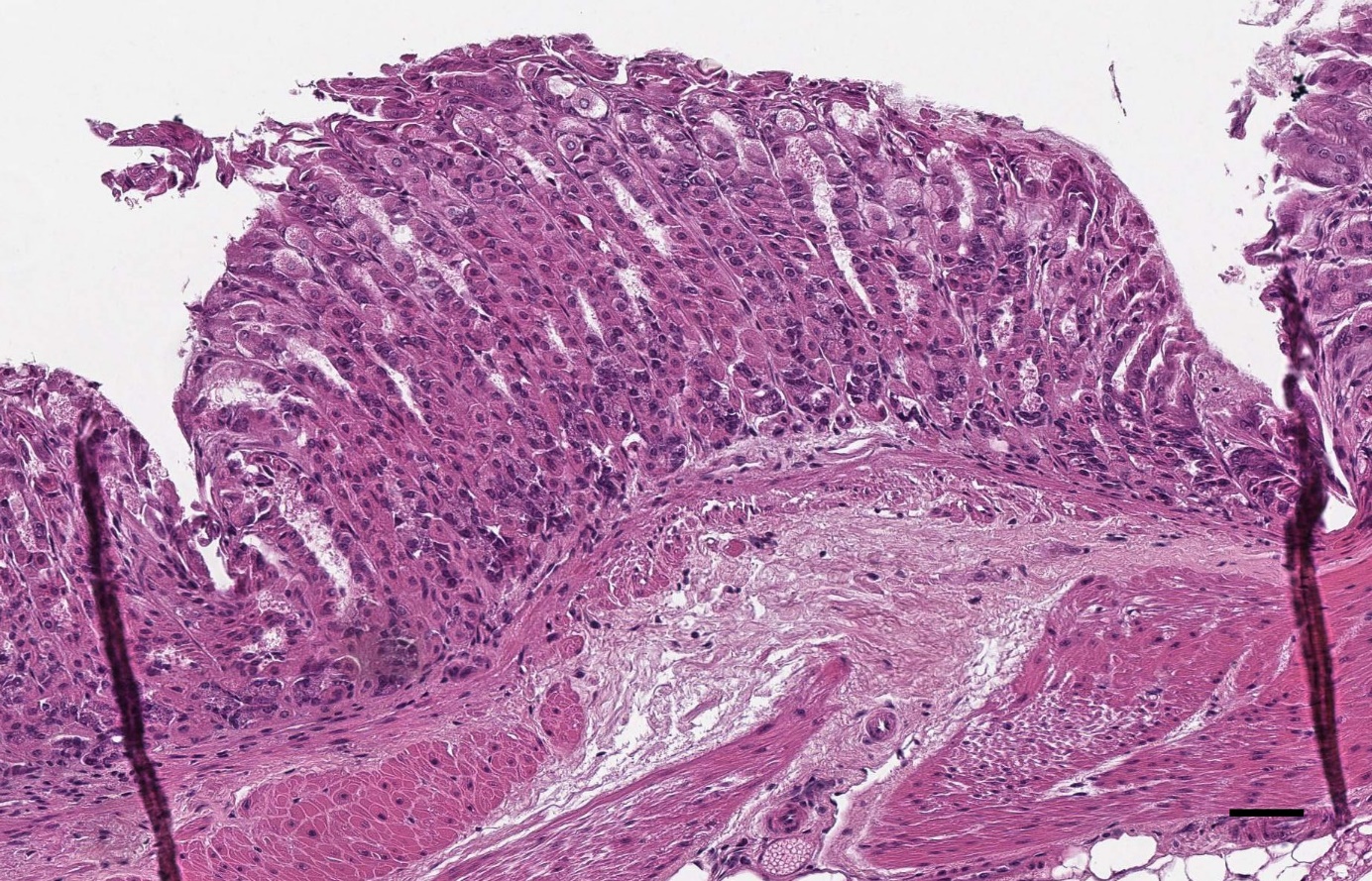
Figure S7: non-infected mice at M12 (scale bar: 100µm)
